# Characterizing Multifunctional Mesoporous Cerium Silicate Nanoparticles for Potential Use in Bioactive Dental Materials: A Proof-of-Concept Study

**DOI:** 10.3390/ma19112197

**Published:** 2026-05-23

**Authors:** Robert S. Jones, Taruna Singh, Isha Mutreja, Dhiraj Kumar

**Affiliations:** 1 Division of Pediatric Dentistry, Department of Developmental & Surgical Sciences, School of Dentistry, University of Minnesota, Minneapolis, MN 55455, USA; 2 Department of Chemistry, Hansraj College, University of Delhi, Delhi 110007, India; tarunasingh@hrc.du.ac.in; 3 IMpact Biomaterials Lab, Division of Restorative Science, Minnesota Dental Research Center for Biomaterials and Biomechanics (MDRCBB), School of Dentistry, University of Minnesota, Minneapolis, MN 55455, USA; 4 Division of Basic Sciences, Department of Diagnostic and Biological Sciences, School of Dentistry, University of Minnesota, Minneapolis, MN 55455, USA

**Keywords:** dentistry, dental resin, biomaterials, nanoparticles, biocompatibility, polyphosphates

## Abstract

(1) Background: Cerium silicate (CeSi) nanoparticles (NPs) have potential as a restorative filler particle with multifunctional properties to improve longevity. To increase the biological activity, these nanoparticles can be fabricated with ultrasmall pores (mesoporous) (MPCeSi-NP) that can be loaded with a polyphosphate inhibitor, such as gallein. (2) Methods: MPCeSi-NPs were custom-synthesized with a microemulsion method, using cetyltrimethylammonium bromide (CTAB) as a template for self-assembly. Biocompatibility with oral keratinocytes/fibroblasts was tested, with the addition of examining the biomineralization potential with human bone-marrow-derived mesenchymal stromal cells (BM-MSCs). MPCeSi-NP, loaded with gallein, was tested against *Rothia dentocariosa* (*Rd*). MPCeSi-NP was added to a resin matrix of triethylene glycol dimethacrylate (TEGDMA) and Bisphenol A-glycidyl methacrylate (BisGMA) with subsequent mechanical properties evaluation. (3) Results: MPCeSi-NPs had high biocompatibility with oral keratinocytes and fibroblasts, especially at concentrations below 300 µg/mL. MPCeSi-NPs induced the biomineralization of BM-MSCs. Higher cerium levels increased mineralization. MPCeSi-NP had weak antimicrobial activity against *Rd*. At 1% wt, MPCeSi-NPs did not reduce the polymerization potential and mechanical properties of a TEGDMA:BisGMA polymer material, with controlled release of gallein in a simulated degradation model. (4) Conclusions: MPCeSi-NPs are highly biocompatible and bioinductive and have the potential to improve the biological response of current restorative materials.

## 1. Introduction

Dental resin restorations, particularly those repairing cervical class V carious lesions, have progressive failure during their lifetime [1,2]. Secondary caries development, driven by acidogenic bacteria within the dental plaque microbiome at the interface of resin-based materials, requires surgical re-intervention and often necessitates aggressive therapy, such as endodontic treatment or extraction [3,4,5]. In addition to marginal and localized demineralization, dental restorations can induce inflammation of periodontal tissues [6]. Inflammation may be exacerbated by leachable compounds within resin composite systems [7,8]. To improve restoration longevity, there is a clear need to enhance the biocompatibility and bioactivity of the current materials via tailoring the chemistry of the base material or by adding a component to modify the overall surface and material properties.

One approach to improving restorative resin materials and dental resin adhesives is to add nanoparticles (NPs) that exert multifunctional effects on plaque biofilms and host cells [9]. NPs have broad applications in dentistry and, when incorporated within dental biomaterials, can exhibit multifunctional properties [10,11]. Multifunctional properties can also be achieved by combining NPs that improve mechanical properties with NPs that provide bioactive properties [12]. The list of candidate NPs being currently investigated in dentistry is expansive, with several literature reviews highlighting potential advantages and limitations of NP are in development [9,10,13,14]. One of the most differentiating factors between NP types may be potential biocompatibility and systemic safety. Cerium silicate (CeSi) nanoparticles (NPs) have demonstrated high biocompatibility and are considered systemically safer than cerium oxide NP [15]. CeSi-NPs have the potential to improve the physical and mechanical properties of dental restorative materials and provide inherent nanozyme effects [16,17]. CeSi-NP can have catalase-like activity and scavenge free radicals, at a near neutral pH [18]. This antioxidant property has been linked to the protection of dental stem cells and the promotion of osteoblastic differentiation [17,19]. Interestingly, under weakly acidic conditions, these nanoparticles exhibit pro-oxidative properties, generating highly reactive oxygen species that are potentially antimicrobial [9,18]. Under weakly acidic conditions, catalase activity is suppressed. CeSi-NPs exhibit dual anti- and pro-oxidant activity and may have beneficial effects when used in the oral cavity, which is often exposed to damaging acidic conditions.

To improve the nanozyme activity of CeSi-NP, these nanoparticles can be fabricated with the addition of ultrasmall pores (mesoporous) that increase the surface area to volume ratio [20]. The mesoporous structure can increase nanozyme activity and improve biocompatibility [21,22]. The NP pores can also be loaded with exogenous compounds [23], making CeSi-NP a potential nano-carrier within a resin restorative or adhesive material.

In the proposed work, the loading of nano-additives into mesoporous cerium silicate (MPCeSi) nanoparticles (NPs) will be examined to determine whether these CeSi-NPs can be synthesized to slowly release an additive molecule that induces or attenuates specific biochemical pathways, thereby guiding bacterial cell responses. The demonstrative additive gallein will be studied. Gallein, an anti-polyphosphate molecule, has been shown to target the metabolism of a cariogenic bacterium, *Rothia dentocariosa* [24,25].

The proposed study aimed to examine the broad potential of MPCeSi-NP in restorative dentistry. The biocompatibility of MPCeSi-NP with oral keratinocytes and fibroblasts was tested, with the addition of testing the biomineralization potential with human bone-marrow-derived mesenchymal stromal cells (BM-MSCs). The proposed work hypothesized that MPCeSi-NP would exhibit antimicrobial activity against *Rothia dentocariosa*. In addition, MPCeSi-NP was incorporated into a resin matrix of triethylene glycol dimethacrylate (TEGDMA) and Bisphenol A-glycidyl methacrylate (BisGMA), highlighting its potential application in restorative dentistry as a nano-additive (gallein-loaded) to attenuate bacterial cell proliferation and induce tooth mineralization when/if needed. The study hypothesized that the addition of MPCeSi-NP (1% wt) would not affect the mechanical properties of the resin matrix. Lastly, the study examined the potential of MPCeSi-NP as a nanocarrier for delivering a model additive, gallein.

## 2. Materials and Methods

### 2.1. Synthesis and Analysis of Mesoporous Cerium Silicate Nanoparticles (MPCeSi-NPs)

Nanoparticle synthesis was based on a previously optimized and published method for the synthesis of calcium and strontium-based mesoporous nanostructures [20]. The synthesis employed a microemulsion method, using cetyltrimethylammonium bromide (CTAB, SKU—H5882-500 G) as a template for self-assembly in water and liquid ammonia (liq. NH_4_OH, SKU—1054239025) at room temperature. Tetraethyl orthosilicate (TEOS, SKU—131903-250 mL) and cerium nitrate hexahydrate (Ce(NO_3_)_3_.6H_2_O, SKU—238538-500 G) were subsequently added. All chemicals were purchased from MilliporeSigma (St. Louis, MO, USA). For the synthesis of MPCeSi-NPs, a reaction mixture containing 2.2 g of cetyltrimethylammonium bromide (CTAB), 2 mL of ammonia hydroxide, and 200 mL of water were stirred for 30 min in a 500 mL 2-neck round bottom flask, followed by the addition of a pre-calculated amount of cerium nitrate (Formulation 1:10 (0.1:1) (Ce:TEOS mole ratio) and 4:10 (0.4:1) with 10 mL of tetraethyl orthosilicate (TEOS) at room temperature. The reaction was maintained for an additional 4 h at vigorous stirring, after which the mixture was centrifuged at 3000 rpm to collect the NP powder. The NPs were dried overnight at 60 °C in an oven, followed by calcination at 550 °C for 5 h to remove CTAB from the mesoporous Ce:TEOS NPs. A transmission electron microscope (FEI Tecnai Spirit BioTwin TEM; FEI Co., Hillsboro, OR, USA) at the Characterization Facility (CharFac), Nils Hasselmo Hall, University of Minnesota, Minneapolis, MN was used to assess NP size and structure. A Brunauer–Emmett–Teller (BET) nitrogen gas isotherm analysis at University of Minnesota, Department of Chemistry, Minneapolis, MN was used to measure the surface area and pore diameter (mesoporosity) of the prepared NPs using a Quantachrome Autosorb iQ (Anton Paar, Ashland, Virginia, USA) system.

### 2.2. Biocompatibility and Osteogenic Differentiation

Oral human keratinocytes, derived from a non-neoplastic line (OKF6/TERT-2, BWH Cell Culture and Microscopy Core, Boston, MA, USA) and gingival human fibroblasts (CS-201–018, ATCC, Manassas, VA, USA), were used to study the biocompatibility response toward MPCeSi-NPs. Formulations of Ce:TEOS ratios of 1:10 and 4:10 were used at concentrations from 0 to 300 µg/mL. Cultures of oral human keratinocytes were grown using previously established conditions with defined keratinocyte, serum-free media containing 1% penicillin/streptomycin to remove bacterial contamination (Gibco, Thermo Fisher, Waltham, MA, USA) [26]. Fibroblasts were cultured in low-serum media (ATTC, Manassas, VA, USA) containing the same antibiotics. Cells (10,000 cells per well, *n* = 3) were cultured for 24 h in 96-well plates. Metabolic activity (viability) as a measure of cytotoxicity was determined using alamarBlue™ (Thermo Fisher Scientific, Waltham, MA, USA) according to the manufacturer’s instructions. Fluorescence intensity was measured at 540/590 nm excitation/emission wavelengths on a multimode microplate reader (Synergy HT, BioTek, Miami, FL, USA).

To evaluate the osteogenic differentiation and matrix mineralization capability of MPCeSi-NPs, the NPs were incubated with human bone-marrow-derived mesenchymal stromal cells (BM-MSCs) [20,27,28,29]. The mesoporous cerium silicate NPs at lower concentrations, 3 and 15 µg/mL (supporting cell viability in fibroblast and oral keratinocytes), were exposed to BM-MSCs (20,000 cells per well, *n* = 3) up to 28 days in osteogenic media in a 24-well plate. The media and NPs suspensions were refreshed at predetermined time points. On day 28, the cells were washed with a phosphate buffer saline (PBS, 1× dilution) and water. Cells were then incubated with 40 mM Alizarin Red solution for 10 min, followed by washing the cells in each well with deionized water until the washing solutions were clear. Thereafter, to perform a quantitative analysis of the cell-mediated stain/matrix mineralization, the stained mineralized matrix was dissolved by adding 10% glacial acetic acid (500 µL) to each well, followed by a 30-min incubation. The optical density (absorbance) at 405 nm was measured using a microplate reader (Synergy, HT, BioTek, USA) by transferring 300 µL of suspension from each well of a 96-well plate [20,30].

### 2.3. Bacterial Response Towards MPCeSi-NPs

*Rothia dentocariosa* (*Rd*, ATCC 17931, USA) was taken from a frozen stock and cultured on the Brain Heart Infusion (BHI) media–agar (MilliporeSigma (53,286–500 g) for 24–48 h. Single colonies were transferred into 5.0 mL BHI broth and incubated overnight under aerobic conditions with continuous shaking (180 rpm) in 15 mL centrifuge tubes. Overnight cultures were diluted to ~0.15 OD in fresh BHI medium, incubated with Ce:TEOS formulations at 1:10 and 4:10 ratios at concentrations ranging from 0 to 300 µg/mL, and cultured for 24 h in 15 mL centrifuge tubes (*n* = 3). In total, 1 mL of aliquots was removed, and OD at 600 nm was measured to obtain bacterial growth curves.

### 2.4. Properties of MPCeSi-NPs Filled Resin Matrix

Following the NPs characterization, standardized discs (radius = 5 mm, depth = 2.2 mm) were prepared using a previously published fabrication method [31]. Discs were made with triethylene glycol dimethacrylate (TEGDMA) and Bisphenol A-glycidyl methacrylate (BisGMA). TEGDMA:BisGMA discs mixed (60:40% *w*/*w*) for 3 days, initially in a rotating mixer. For samples in which MPCeSi-NPs were added (1% *w*/*w*), particles were added during mixing, followed by final sonication for 10 s in an ice bath to prevent overheating the mixture. Both formulations of the Ce:TEOS ratio of 1:10 and 4:10 were examined. The monomer and photo/co-initiator (camphorquinone—0.5%, EDMAB—1.2%, and (Ph_2_I)^+^(PF_6_)^−^—1.2%) mixture was placed in a PTFE mold and cured for 40 s at 1000 mW/cm^2^ power using Ultradent VALO cordless LED light source at 385–515 nm (Ultradent, VALO, South Jordan, UT, USA).

To measure the polymerization efficacy of the TEGDMA:BisGMA discs, the degree of conversion (DC) of the monomers was measured using an FTIR-ATR analysis. Polymer discs were stored for 24 h in airtight glass containers at room temperature before mechanical, physical, and chemical characterization. The bottom part of the polymer discs was brought into contact with the diamond crystal, and force was applied to improve the signal-to-noise ratio. A Nicolet iS50 Fourier Transformation Infrared Spectroscopy (FTIR) system in Attenuated Total Reflectance (ATR) mode was used in transmittance mode (2 cm^−1^ resolution and 25 scans for each sample) over a 400–4000 cm^−1^ range. The acquired data were converted to absorbance (OMNIC, Thermo Fisher, Waltham, MA, USA). The absorbance data were used to calculate the degree of conversion or polymerization using a previously reported equation [31].

The micro hardness of polymer discs, with and without MPCeSi-NPs, was assessed using a Vickers Hardness Tester (Buehler Micromet II, Buehler Ltd., Lake Bluff, IL, USA). The microhardness tester used a diamond pyramid micro-indenter with an angle of 136° between the opposing faces. The microhardness test used a 100 g applied load, held for 20 s to complete the measurement. The diametral tensile strength (DTS—kg*f/cm^2^) of the polymer discs was measured using a material testing system with an applied load of up to 5 kN (858 Mini Bionix^®^ II, MTS, Eden Prairie, MN, USA). Vickers hardness (HV) and DTS were obtained using previously published equations [31].

### 2.5. Nano-Additive Loading of MPCeSi-NPs and Release Kinetics

To evaluate the nano-additive loading potential of the mesoporous NPs, gallein was loaded into MPCeSi-NPs. Examination was conducted on NPs alone and within the TEGDMA-BisGMA matrix. A 1 mg/mL stock solution of gallein (2.443 mM) in H_2_O was prepared. The absorbance of gallein at 277 nm was used to develop a gallein absorbance standard curve drawn for different dilution standards using a microplate reader (Synergy, HT, BioTek, USA). NPs (10 mg) were weighed and mixed with 1 mL of gallein solution (1 mg/mL, 2.443 mM) for 24 h to facilitate gallein loading. NPs were then washed with water (×2) by centrifugation. The supernatant was carefully removed, and 1 mL of DI water was added. The mixture was incubated at 37 °C to promote gallein release. The MPCeSi-NPs were examined over a 24-h period. TEGDMA-BisGMA discs containing 1% wt of MPCeSi-NPs were examined for gallein release over 5 weeks in 0.5 units/mL of a cholesterol esterase (CE) buffer (1.0 mL) solution. At specified time intervals, the Eppendorf tubes were centrifuged, the supernatant was collected, measured at 277 nm, and replaced with 1 mL of CE (0.5 units/mL) buffer. MPCeSi-NP (1:10) were loaded with gallein and incorporated into the TEGDMA-BisGMA resin matrix. The discs were stored in aqueous solutions of cholesterol esterase (0.5 units/mL) and renewed weekly for 5 weeks.

### 2.6. Statistical Analysis

Each measurement described above was performed in triplicate with independent samples. Comparisons across groups were performed using ANOVA, with Tukey’s multiple comparisons. In cases where formulations were compared to a control, such as the unfilled TEGDMA:BisGMA, a post hoc analysis was performed with a Dunnett’s multiple comparison test. An analysis was conducted using GraphPad Prism version 8.0.2 software. The 95% confidence intervals have been used as error bars in all the graphs/data sets. The asterisk representation is as follows: non-significant (ns) for *p* > 0.05, * for *p* ≤ 0.05 (a), ** for *p* ≤ 0.01 (b), *** for *p* ≤ 0.001 (c), and **** for *p* ≤ 0.0001 (d).

## 3. Results

**Figure 1:**
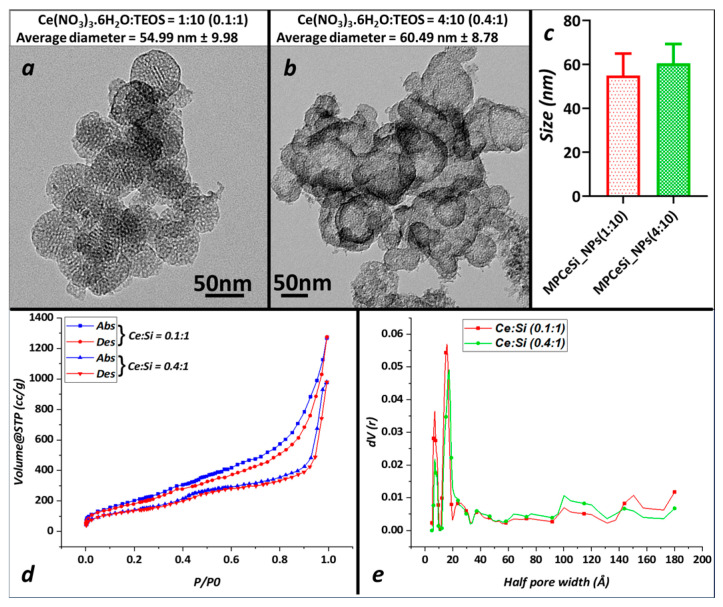
Transmission electron microscopy of MPCeSi-NPs. (**a**) Ce:TEOS-1:10 (93,000× magnification). (**b**) Ce:TEOS 4:10 ratio (73,000× magnification). (**c**) Average particle sizes for the NPs were measured with Ce:TEOS ratios of 1:10 and 4:10 using TEM images; approximately 100 particles were measured diagonally. Images (**d**,**e**) from the BET analysis showing the absorption and desorption nitrogen isotherm and pore size distribution graphs for both nanoparticle types. No statistical difference was found between the two particle types.

**Figure 2:**
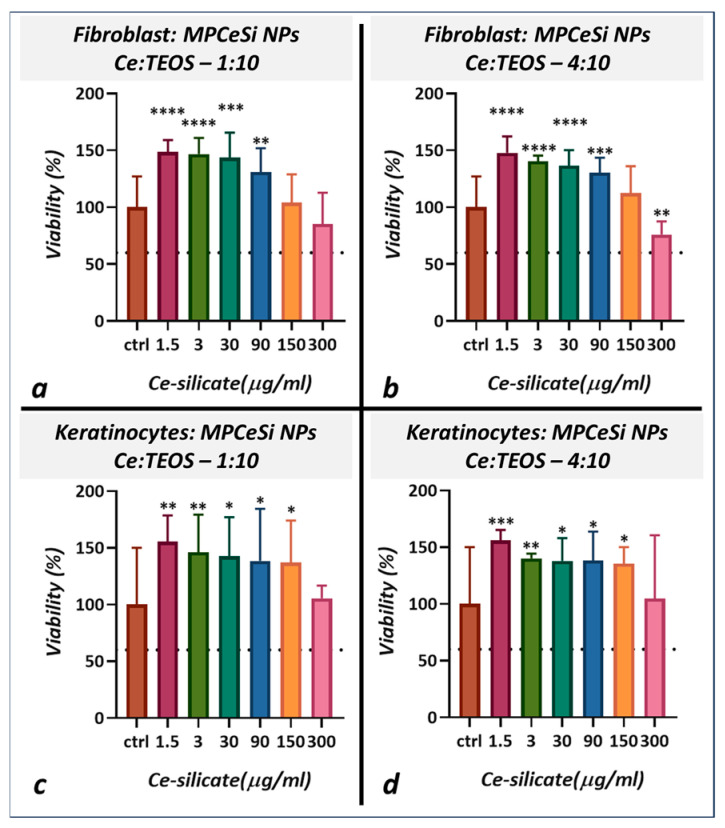
Fibroblast viability, measured by alamarBlue™ assay, was examined 24 h after continuous exposure to MPCeSi-NPs with (**a**) Ce:TEOS molar ratio of 1:10 and (**b**) 4:10. Oral keratinocyte viability with (**c**) 1:10 and (**d**) 4:10. * *p* ≤ 0.05, ** *p* ≤ 0.01, *** *p* ≤ 0.001, and **** *p* ≤ 0.0001.

The synthesis of MPCeSi-NP was performed using cerium nitrate and tetraethyl orthosilicate as precursors, with Ce:TEOS molar ratios of 1:10 and 4:10. Post NPs synthesis and calcination, the NPs were examined using transmission electron microscopy (TEM) for particle size and shape (Figure 1). A consistent spherical particle size was achieved through the synthesis. The nanoparticle diameter increased from an average of 54.99 ± 9.98 nm (1:10) to 60.49 ± 8.78 nm (4:10), although this change was not significantly different. However, electron microscopy also revealed some aggregates with increased cerium concentration. An increase in cerium content within MPCeSi nanostructures reduced porosity, as indicated by a BET nitrogen gas isotherm analysis. For NPs, the total surface area was 536.78 ± 166.67 m^2^/g (1:10) and 426.45 ± 126.55 m^2^/g (4:10) with pore diameters of 3.2 ± 0.43 nm and 3.7 ± 0.83 nm, respectively. Statistically, there was no difference in size, surface area, and pore diameters for these two particle types. To evaluate the biocompatibility of MPCeSi-NPs with gingival fibroblasts and oral keratinocytes, metabolic activity was assessed, and a concentration-dependent response was observed (Figure 2). MPCeSi-NPs at a 300 µg/mL concentration in the cell culture statistically reduced cell viability of fibroblasts versus the no-treatment control for the 4:10 composition. This decrease in cell viability was also observed at the 300 µg/mL concentration against oral keratinocytes, although the difference was not statistically significant. For the 1:10 composition, MPCeSi-NPs at 300 µg/mL showed a non-significant trend toward decreased cell viability. However, at lower concentrations (1.5–30 µg/mL) of NPs (for both compositions), cell viability was higher than in the no-treatment controls, suggesting an upregulation of metabolic activity in response to NPs exposure. However, the nanoparticles with a 4:10 (Ce:Si) ratio showed either the same or slightly lower viability % compared to the same concentration at 1:10 (Ce:Si).

**Figure 3:**
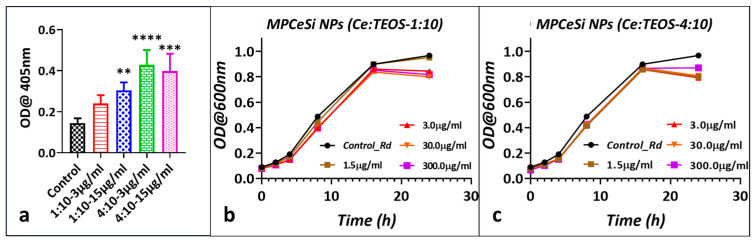
(**a**) Bone-marrow-derived mesenchymal stromal cells (BM-MSCs) were incubated for 28 days with exposure to MPCeSi-NP with Ce:TEOS ratios of 1:10 and 4:10. Matrix mineralization in BM-MSCs was measured with Alizarin red staining, absorbance at 405 nm (**b**,**c**), and optical density at 600 nm over 24 h of bacterial growth curves of *Rothia dentocariosa* (*Rd*) in BHI broth solution continuously exposed to MPCeSi-NP with (**b**) Ce:TEOS ratios 1:10 and (**c**) 4:10 at various concentrations (µg/mL). Mean values are plotted without error bars for readability. A 24-h analysis with 95% confidence intervals is shown in Supplementary Figure S1. ** *p* ≤ 0.01, *** *p* ≤ 0.001, and **** *p* ≤ 0.0001.

For the MPCeSi-NPs that were incubated for 28 days with human bone-marrow-derived mesenchymal stromal cells, matrix mineralization was observed in both the 3 and 15 µg/mL cultures for both nanoparticle types (Figure 3a). At 3 µg/mL, only the higher cerium content (4:10) Ce:TEOS NP showed greater mineralization potential than the untreated controls. At 15 µg/mL, both the 1:10 and 4:10 MPCeSi-NP formulations initiated mineralization in BM-MSCs after 28 days in the osteogenic media. Furthermore, the effect of MPCeSi-NP on the *Rd* bacterial cells is shown in Figure 3b,c. For both the 1:10 and 4:10 Ce:TEOS formulations, MPCeSi-NP did not exhibit an immediate antimicrobial effect at concentrations up to 300 µg/mL. MPCeSi-NP did not affect the duration of the initial lag phase, the timing of the exponential growth phase, or its duration. Between 16 and 24 h, when planktonic bacteria of *Rd* were transitioning into a stationary growth phase in the control, bacterial cultures exposed to MPCeSi-NPs at concentrations up to 300 µg/mL exhibited predominantly declining optical densities. Cultures exposed to MPCeSi-NPs entered the declining death phase earlier. The difference in optical density was significantly different at 24 h between the untreated control samples and bacteria exposed to MPCeSi-NP (Supplementary Figure S1).

**Figure 4:**
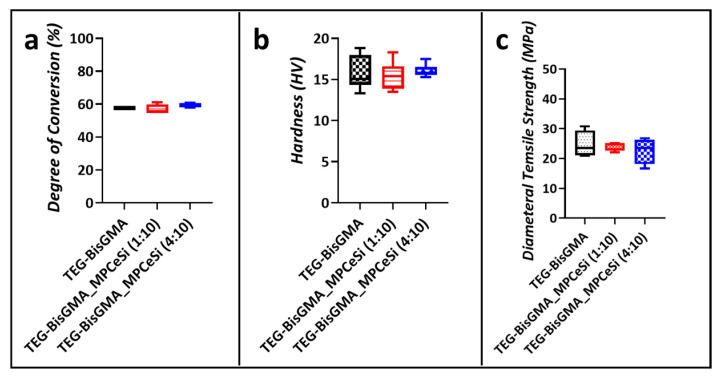
(**a**) Degree of conversion, (**b**) hardness, and (**c**) diametral tensile strength of resin matrix discs with TEGDMA:BisGMA with the addition of mesoporous cerium silicate nanoparticles (MPCeSi-NP, 1% wt) at Ce:TEOS ratios of 1:10 and 4:10. No significant differences (*p* > 0.05) were found between groups.

For the initial chemical characterization and material assessment of all groups, the prepared discs were analyzed using FTIR, and the absorbance values at 1608 cm^−1^ and 1637 cm^−1^ were used to calculate the degree of conversion (DC) or polymerization (Figure 4a), which is a measure of the number of polymerized methacrylate groups. The addition of MPCeSi-NP did not deleteriously decrease the DC or the hardness of the TEGDMA:BisGMA polymer discs at both 1:10 and 4:10 formulations. Gallein also did not deleteriously impact the DC or hardness of the polymer discs (Supplementary Figure S2). The hardness and diametral tensile strength (Figure 4b,c) of the TEGDMA:BisGMA polymer discs were comparable between the pristine polymer discs and the addition of the MPCeSi-NPs.

**Figure 5:**
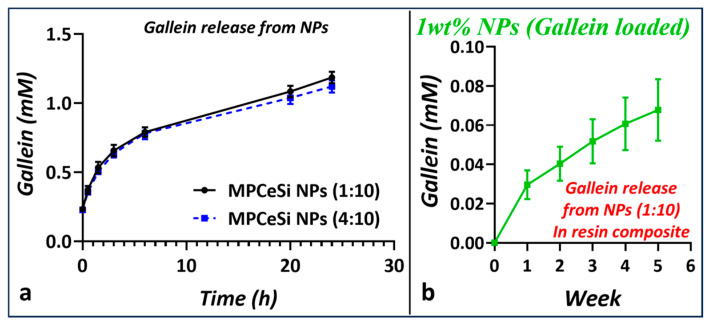
After nano-additive loading of MPCeSi-NP, release of gallein from (**a**) MPCeSi-NP Ce:TEOS ratios of 1:10 (solid) and 4:10 (dashed) after 24 h, and (**b**) TEGDMA:BisGMA resin discs with MPCeSi-NP (Ce:TEOS 1:10) loaded with Gallein (50 µg/mL)—1:1 after 5 weeks.

To assess gallein release kinetics, a standard curve at 277 nm was constructed by plotting concentration versus absorbance (Supplementary Figure S3a). For gallein-loaded MPCeSi-NPs, both 1:10 and 4:10 concentrations demonstrated a sustained-release profile of gallein over 24 h, with no difference between the two formulations, as shown in Figure 5a. For MPCeSi-NP mixed into TEGDMA:BisGMA polymer discs, gallein was released over the course of 5 weeks in the presence of a 0.5 units/mL cholesterol esterase enzyme, with a lower concentration than seen with the NP alone, but the release was steady over 5 weeks as shown in Figure 5b. While MPCeSi-NP loading at both 1:10 and 4:10 altered the translucency of the resin matrix, it did not substantially change the visual color appearance (Supplementary Figure S3b). This contrasts with gallein loading, which changes the color to a brownish tone.

## 4. Discussion

The results of the study support the multifunctional attributes of MPCeSi-NP at Ce:TEOS molar ratios of 1:10 and 4:10. The study examined MPCeSi-NPs both in the NP form and in a resin matrix. The results suggested that MPCeSi-NPs could be used in various dental products. Potential uses include within resin-matrix composite systems, superficial resin coatings, and resin dental cements that can promote bone and gingival health of subgingival resin restorations. Various mechanisms support the use of MPCeSi-NPs in pulp capping materials, where nano-additive loading could provide antimicrobial activity via polyphosphate uptake inhibition, thereby reducing secondary caries formation.

The study investigated MPCeSi-NPs synthesized using two different molar ratios of cerium nitrate added to tetraethyl orthosilicate (TEOS) in the presence of liquid NH_3_ and the CTAB template. The reaction mixture underwent polycondensation in the presence of liquid NH3, forming a metal–oxygen–silicon bond around the CTAB template, thereby imparting mesoporous structure within the nanoparticles. The purpose of this investigation was to determine how the higher concentration of cerium (4:10) may change the particles and their bioactivity compared to a lower concentration (1:10). The higher concentration did not substantially alter the particle size or surface area. Higher concentrations did alter biocompatibility, particularly with gingival fibroblasts. By increasing the cerium concentration, some aggregation was observed. A similar observation has been reported for Cu-Mn-Ce metal silicate nanostructures [32]. However, we did not observe a significant change in pore diameter and surface area. This also suggested the need to optimize the synthesis method. Overall, the material properties of both formulations were equivalent, with neither offering an advantage in terms of mechanical properties. This is a direct effect of MPCeSi-NPs being relatively small and not interfering with the polymerization process or the final degree of conversion of the polymer discs. In the present study, a dual-polymer system was used, in which TEGDMA, a hydrophilic monomer that imparts flowability to the dental formulation, constitutes approximately 40% of the formulation. BisGMA is a hydrophobic monomer used as a structural monomer that adds aesthetics, strength, reduces polymer shrinkage, and improves hydrolytic stability [33]. MPCeSi-NP was compatible with the TEGDMA:BisGMA polymer system, and importantly, did not interfere with the camphorquinone-based photoactivator reaction. Additional studies should examine MPCeSi-NPs in other resin formulations and in other filler systems. Future studies are also needed to examine the distribution of MPCeSi-NPs within the resin matrix.

The main advantage of using a 4:10 concentration was seen in the biomineralization mechanism, where lower concentrations of the NP could induce more biomineralization from human bone-marrow-derived mesenchymal stromal cells (BM-MSCs). Both formulations, 1:10 and 4:10, induced bone mineralization relative to the untreated control. Future studies are needed to examine MPCeSi-NPs in resin composite materials or resin adhesive coatings to repair or maintain periodontal health around subgingival restorations, crowns, or dental implants. The current study is limited to examining the effect of MPCeSi-NP in BM-MSC cell cultures. More work is needed to examine the bioinductive potential in animal models and complex systems, and to evaluate immune responses that may attenuate bioinductive properties.

Both 1:10 and 4:10 Ce:TEOS formulations were found to have relatively weak intrinsic anti-microbial activity. The activity was observed in the later stages of the growth curves of *Rothia dentocariosa* (*Rd*). Planktonic colonies of *Rd* entered the stationary and death phase earlier than the untreated controls. This was observed with both 1:10 and 4:10 Ce:TEOS formulations. Further work is needed on assessing the mechanisms underlying the antimicrobial effects. Previous studies have demonstrated that *Rd* cultures will rapidly metabolize the available carbohydrates in the first 8–9 h and produce acidic cultures (pH = 5.74) [24]. Later in the growth phase of *Rd*, there will be a subsequent rise in pH, toward a weakly acidic condition, from the shift of metabolism toward protein catabolism after the available carbohydrates are depleted [24]. This previous work demonstrated that after 19 h, the pH within the cultures rose above 6.0, toward a pH of 6.21 after 24 h of growth [24]. The pH is in the range where MPCeSi-NPs have pro-oxidative properties [9]. In this weakly acidic environment, catalase activity is suppressed, and MPCeSi-NP produce reactive oxygen species that are highly reactive and potentially antimicrobial [9,18]. Future studies on *Rd* and other bacterial species implicated in secondary caries and gingival inflammation can investigate this hypothesis and other potential antimicrobial effects on bacterial growth.

The novel mesoporous cerium silicate particles can be filled with nano-additives. Future studies can investigate a range of nano-additives and their effects on secondary caries and periodontal health. In the present study, gallein, a xanthene-based compound, was investigated as a representative nano-additive for use as a bioactive liner in the interior of resin restorations, within core build-ups, or integrated into crown cements. Since gallein has deleterious effects on the tooth-colored shade of resin discs, future work is needed to examine the use of opacifiers or pre-conditioning compounds to improve clinical aesthetics. These efforts may be worthwhile given that gallein is a potent inhibitor of polyphosphate synthesis. Oral bacteria, such as *Rd*, can accumulate high concentrations of phosphate and synthesize long polyphosphate (polyP) chains [24,25,34]. PolyP provides *Rd* with a reservoir of inorganic phosphate that can serve as an energy reserve and is linked to stress responses and cell survival [35,36]. Gallein has been shown to inhibit this virulence factor [24,37]. The present study investigated whether MPCeSi-NP can serve as a delivery particle for gallein. The present study demonstrated that MPCeSi-NPs could be loaded with gallein and achieve sustained release. The present work also demonstrated that MPCeSi-NP, preloaded with the nano-additive of gallein, had a sustained release in a clinically relevant TEGDMA:BisGMA polymer degradation model. The present study used cholesterol esterase to simulate the natural esterases found in saliva and in bacteria in the oral cavity [38,39]. Gallein was slowly released from this system. In the present study, the first-week release (29.6 µM) was equivalent to the gallein concentration (25 µM) used to modulate bacterial cell responses and attenuate phosphate uptake and polyP synthesis [24].

As a proof-of-concept study, the limitations include a lack of comprehensive analysis on the effect of MPCeSi-NP, with and without gallein, on polyphosphate update in the oral microbiome and secondary caries formation. Future studies can examine how MPCeSi-NP with and without gallein may affect the oral microbiome, secondary caries formation, and material degradation.

## 5. Conclusions

Novel mesoporous cerium silicate nanoparticles (MPCeSi-NP) were synthesized in this study and had multifunctional properties that have the potential to improve dental restorative materials.

- MPCeSi-NPs had high biocompatibility with oral keratinocytes and fibroblasts, especially at concentrations below 300 µg/mL.
- MPCeSi-NPs induced the biomineralization of human bone-marrow-derived mesenchymal stromal cells (BM-MSCs). Higher cerium levels increased mineralization.
- MPCeSi-NPs had weak antimicrobial activity against *Rothia dentocariosa*.
- At 1% wt, MPCeSi-NPs did not reduce the polymerization potential and mechanical properties of a TEGDMA:BisGMA polymer material.
- MPCeSi-NPs have the potential to be loaded with nano-additives, such as gallein, and provide controlled release.

## Data Availability

These data were derived from the following resources available in the public domain through the Data Repository for the University of Minnesota (DRUM): https://hdl.handle.net/11299/280655, accessed on 22 05 2026.

## Supplementary Materials

The following supporting information can be downloaded at: https://www.mdpi.com/article/10.3390/ma19112197/s1, Supplementary Figure S1: Optical density at 600 nm at 24 h of bacterial growth curves of *Rothia dentocariosa* in BHI broth solution continuously exposed to MPCeSi NP with (a) Ce:TEOS 1:10 and (b) 4:10 at various concentrations (µg/ml). Mean values and 95% Confidence intervals are shown. **** for p ≤ 0.0001; Supplementary Figure S2: (a) Degree of conversion and (b) hardness of resin matrix discs with TEGDMA:BisGMA with the addition of mesoporous cerium silicate nanoparticles (MPCeSi NP, 1% wt) and nano-additive loading of MPCeSi with gallein. * for p ≤ 0.05; and Supplementary Figure S3: (a) Standardize Curve of gallein at 277 nm that plots concentration of the nano-additive gallein with absorbance (b) Photos of resin matrix discs with TEGDMA:BisGMA with mesoporous cerium silicate nanoparticles (MPCeSi NP, 1% wt). Nano-additive loading of the mesoporous particle with Gallein alters the optical properties substantially.

## Abbreviations

The following abbreviations are used in this manuscript:

ANOVA	Analysis of Variance
BET	Brunauer–Emmett–Teller
BHI	brain heart infusion
BisGMA	Bisphenol A-glycidyl methacrylate
BM	bone marrow
CeSi	cerium silicate
CTAB	cetyltrimethylammonium bromide
DI	deionized
DTS	diametral tensile strength
FTIR-ATR	Fourier-Transform Infrared Spectroscopy- Attenuated Total Reflectance
MPa	megapascals
MPCeSi	mesoporous cerium silicate
MSC	mesenchymal stromal cells
NP	nanoparticles
*Rd*	*Rothia dentocariosa*
TEGDMA	triethylene glycol dimethacrylate
TEOS	tetraethyl orthosilicate
Wt	weight
