# Supplementary material for: Characterizing Multifunctional Mesoporous Cerium Silicate Nanoparticles for Potential Use in Bioactive Dental Materials: A Proof-of-Concept Study
Source: Materials (Basel). 2026 May 23;19(11):2197. doi: 10.3390/ma19112197 (PMC13257735; doi:10.3390/ma19112197)
Supplement: Supplementary file 1 [file materials-19-02197-s001.zip › materials-4294238-supplementary.pdf]

Journal of
Functional Biomaterials

MDPI

Supplementary Material

Characterizing Multifunctional Mesoporous Cerium Silicate Nanoparticles for Potential Use in Bioactive Dental Materials: A Proof-of-Concept Study

Robert S. Jones [1,*], Taruna Singh [2], Isha Mutreja [3,†] and Dhiraj Kumar [4,*]

[1] Division of Pediatric Dentistry, Department of Developmental & Surgical Sciences, School of Dentistry, University of Minnesota, Minneapolis, MN, 55455 USA
[2] Department of Chemistry, Hansraj College, University of Delhi, Delhi, 110007 India; tarunasingh@hrc.du.ac.in
[3] IMpact Biomaterials Lab, Division of Restorative Science, Minnesota Dental Research Center for Biomaterials and Biomechanics (MDRCBB), School of Dentistry, University of Minnesota, Minneapolis, MN, 55455 USA
[4] Division of Basic Sciences, Department of Diagnostic and Biological Sciences, School of Dentistry, University of Minnesota, Minneapolis, MN, 55455 USA
* Correspondence: rsjones@umn.edu (R.S.J.); haridhiraj@gmail.com (D.K.)
† Deceased author.

1. Supplementary Material

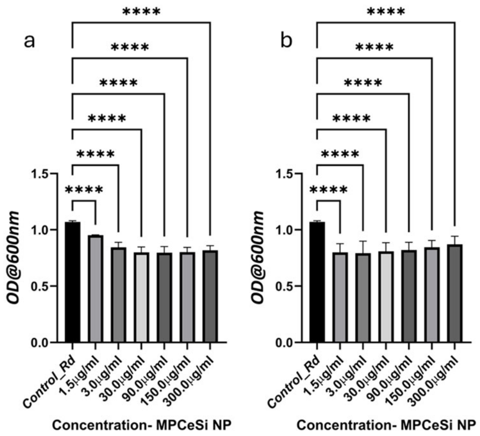

Supplementary Figure S1. Optical density at 600 nm at 24 hours of bacterial growth curves of *Rothia dentocariosa* in BHI broth solution continuously exposed to MPCeSi NP with a) Ce:TEOS 1:10 and b) 4:10 at various concentrations (μg/ml). Mean values and 95% Confidence intervals are shown. **** for $p \leq 0.0001$

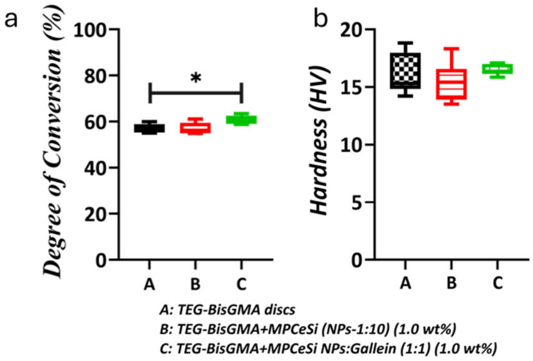

Supplementary Figure S2 a) Degree of conversion and b) hardness of resin matrix discs with TEGDMA:BisGMA with the addition of mesoporous cerium silicate nanoparticles (MPCeSi NP, 1% wt) and nano-additive loading of MPCeSi with gallein. * for $p \leq 0.05$

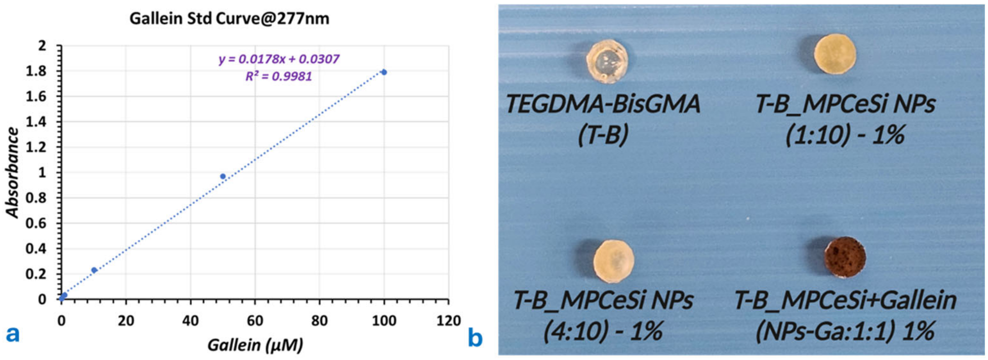

Supplementary Figure S3 a) Standardize Curve of gallein at 277 nm that plots concentration of the nano-additive gallein with absorbance b) Photos of resin matrix discs with TEGDMA:BisGMA with mesoporous cerium silicate nanoparticles (MPCeSi NP, 1% wt). Nano-additive loading of the mesoporous particle with Gallein alters the optical properties substantially.

Disclaimer/Publisher's Note: The statements, opinions and data contained in all publications are solely those of the individual author(s) and contributor(s) and not of MDPI and/or the editor(s). MDPI and/or the editor(s) disclaim responsibility for any injury to people or property resulting from any ideas, methods, instructions or products referred to in the content.
